# Validation of the HHHHHMM Scale in the Italian Context: Assessing Pets’ Quality of Life and Qualitatively Exploring Owners’ Grief

**DOI:** 10.3390/ani13061049

**Published:** 2023-03-14

**Authors:** Ines Testoni, Ciro De Vincenzo, Michela Campigli, Aljosha Caregnato Manzatti, Lucia Ronconi, Stefania Uccheddu

**Affiliations:** 1 Department of Philosophy, Sociology, Education, and Applied Psychology (FISPPA), University of Padova, 35131 Padua, Italy; ciro.devincenzo@unipd.it (C.D.V.); aljosha.caregnatomanzatti@studenti.unipd.it (A.C.M.); l.ronconi@unipd.it (L.R.); 2 Sagol Creative Arts Therapies Research Center, Haifa 31905, Israel; 3 San Marco Veterinary Clinic and Laboratory, 35030 Veggiano, Italy; michelacampigli@virgilio.it (M.C.); stefania.uccheddu@sanmarcovet.it (S.U.)

**Keywords:** pet loss, pet grief, pet quality of life, pet bereavement, veterinary support

## Abstract

**Simple Summary:**

Human guardians and companion animals develop special and unique bonds. Therefore, witnessing the terminal illness and subsequent death of a companion animal can be a stressful experience for human guardians. Professionals in the veterinary and psychological sciences can support human guardians through the caring and grieving processes. The aim of this research was to validate in the Italian context the HHHHMM Quality of Life Scale, which is specifically used to help human guardians assess companion animals’ quality of life. To this end, other scales and open-ended questions were adopted to test hypotheses and deepen understanding of the grieving experience. The results confirmed the usefulness of the scale and highlighted important correlations between age, bereavement, and attachment. Further, a thematic qualitative analysis revealed the importance of the relationship between the human guardian and the veterinarian as well as the need for social support after the loss. The findings clearly showed that the bond between a human guardian and a companion animal does not cease after the loss of the animal; rather, it continues in new forms. Overall, the present research confirmed the importance of the veterinary and psychological sciences working together to provide complete support for human guardians.

**Abstract:**

Witnessing a companion animal’s death can be a stressful psychological experience for human guardians, affecting their ability to grieve. The veterinary and psychological sciences offer useful tools for supporting human guardians during their companion animal’s terminal illness. Accordingly, the present study aimed to validate the HHHHMM Quality of Life Scale in the Italian context. The study followed a mixed-methods design and involved 314 participants. The Mourning Dog Questionnaire (MDQ), Lexington Attachment to Pets Scale (LAPS), Pet Bereavement Questionnaire (PBQ), and open-ended questions were adopted to test the research hypotheses and qualitatively explore the grieving experience. The results showed that the model’s fit was partially adequate, with all parameters being significant and over 0.40. Moreover, human guardians’ anger levels were high when their companion animal’s quality of life was poor, and greater levels of grief were associated with higher levels of attachment. Gender differences were observed only with the LAPS, and a negative correlation with age was found with the LAPS and PBQ. A thematic qualitative analysis revealed four themes: continuing bonds, coping strategies, shared moral values, and perceived support. Thus, the research reaffirmed the importance of adequate veterinary and psychological support for human guardians experiencing the loss of companion animals.

## 1. Introduction

Bereavement is a normal psychological experience that is prompted by the death of a significant one [1] and is characterized by complex, multifaceted feelings of grief as well as different mourning practices [2]. In modern times, the ecological cohabitation of humans and other animals has increased to the degree that companion animals represent fundamental members of the domestic space. Indeed, people often develop deep emotional connections with companion animals, and almost all human guardians in Western societies consider them family members [3]. Therefore, recent studies have recognized that human guardians may experience considerable grief over the loss of companion animals [4,5].

Gerwolls and Labott [6] found the grief after the loss of a companion animal and the grief after the loss of a human being to have comparable intensities, whereas Hunt and Padilla [7] stated that the bereavement associated with the loss of a companion animal may even be even greater than that experienced following the death of a person [8]. Moreover, the quality of the relationship and, more specifically, the degree of attachment between a human guardian and a companion animal [9] is a key factor determining the psychological burden the human guardian experiences due to companion animal loss [10]. However, despite the frequency and intensity of the grief that people experience due to companion animal loss, this phenomenon lacks proper social recognition, and there is little opportunity for adequate support [11]. As a matter of consequence, the bereavement process usually assumes the form of disenfranchised grief—that is, grief which is not socially acknowledged and becomes a source of additional suffering for the bereaved [5]. Cordaro [12] argued that this disenfranchised grief has three major causes: considering the bereavement over a companion animal unacceptable, believing that the individual can quickly cope with grief and easily replace the lost companion animal, and not considering the mourning experience as authentic. An eloquent example is the dog–guardian relationship. The literature shows that human guardian–dog relationships provide the same, or even greater, sense of comfort, security, and affection that close human relationships do [3]. Several variables influence people’s experiences upon the loss of their dog, including the nature and quality of their attachment bond, the quality of social support received for the bereavement, and the circumstances under which the death occurred [13]. It should be noted that dogs live much shorter lives than humans. Consequently, human guardians may experience multiple such losses and repeated grief, which may overwhelm their normal ability to adapt.

Since grief due to the loss of a companion animal can be a painful experience, it is of the utmost importance that experts in the psychological and veterinary fields develop targeted psychological instruments to support the bereaved [4,5]. In recent years, there have been increasing calls for veterinarians to provide tools to assess the quality of life (QoL) of elderly or terminally ill companion animals and thereby prevent or ease their suffering. Veterinary medicine now comprises a wide range of technologies and treatment options that make it possible to prolong a companion animal’s life. However, due to the diversity of beliefs that influence veterinarians and human guardians, they are increasingly confronted with ethical dilemmas regarding the appropriateness of available procedures [14]. Continuous progress in veterinary medicine has led to the optimization of decision-making processes affecting the last stages of companion animals’ lives. The veterinarian no longer replaces the client in decision-making but takes on the role of educator and consultant to maintain a balance between animal welfare and client perspective. This mirrors the American Veterinary Medical Association guidelines [15].

Managing a companion animal’s prolonged illness can be complex and time consuming. Recent research shows that the caregiver burden—the distress that emerges when providing care for an individual with an illness [16]—is a frequent occurrence among human guardians and those who care for animals with a chronic or terminal illness [17,18]. This burden is linked to multiple negative psychosocial consequences, including high levels of stress, symptoms of depression and anxiety, and a low quality of life. The importance of understanding clients’ experiences with caring for elderly or sick animals is evident. Caregiver burden appears not only to be a cause of stress for clients but also to play an active role in influencing euthanasia decisions [19]. When clients feel overwhelmed, it is necessary to corroborate their experiences, both in the long-term duration of the companion animal’s illness and in considering euthanasia.

There is currently a lack of studies on QoL assessments for pets at the end of their lives [20]. One such assessment is the HHHHHMM (hurt, hunger, hydration, hygiene, happiness, mobility, more good days than bad) Quality of Life Scale developed by Villalobos [21]. The tool guides people who are attached to their companion animals in considering the state of their companion animal’s health and in assessing whether they are able to provide sufficient care for their sick companion animal. It provides an overall assessment of the daily life of a sick animal suffering from a chronic and progressive disease, evaluating aspects such as pain, hunger, hydration, hygiene, and movement. This is an accessible and easy-to-understand tool for assessing the QoL of terminal animals.

The present research had three specific objectives. The first was to validate the HHHHMM scale in the Italian context. The second was to identify the predictors of QoL based on the characteristics of the companion animal and the impact of the relationship between QoL, attachment, and human guardian/companion animal characteristics and human guardians’ bereavement-related distress. Finally, a qualitative perspective was adopted with the aim of exploring participants’ experiences with companion animal loss.

## 2. Materials and Methods

### 2.1. Aims and Hypotheses

The purpose of the present study was to validate the HHHHMM Quality of Life scale [21] in the Italian context. In addition, the research aimed to identify the predictors of QoL based on the characteristics of the companion animal and its living environment, investigated using questionnaires. Moreover, the research aimed to assess the impact of QoL and attachment together with human guardian and animal characteristics on human guardian distress due to the death of a companion animal. Finally, open-ended questions were qualitatively analyzed to explore participants’ experiences with companion animal loss.

Upon considering other measurement tools (see below), it was hypothesized that human guardians’ levels of emotional attachment to their companion animals constitute the main predictor of bereavement experiences and that the level of emotional attachment mediates the relationship between the human guardian’s gender and bereavement.

### 2.2. Study Design

A mixed-methods design was adopted for this study [22], bringing together the potential of quantitative and qualitative inquiry. This design integrates quantitative and qualitative methods to gain a deeper, more sensitive understanding of the phenomenon under investigation. This paper first presents the quantitative results and then illustrates the qualitative results obtained from the thematic analysis.

The questionnaires for the study were formed from the HHHHMM Quality of Life Scale and three other scales.

1. The first questionnaire used was the Mourning Dog Questionnaire (MDQ) [23], which consists of two sections. The first section is to gather the demographic data of the human guardian, such as gender, age, education level, marital status, occupation, and presence of children. The second section focuses on information related to the deceased dog, specifically age, gender, cause of and time since death, length of cohabitation with the human guardian, and whether the human guardian was living alone at the time of the dog’s death.

Item example: Which of the following best describes the role played by this animal in your relationship? Partner/friend/source of protection/animal from work/family (child)/family (brother/sister)/simply an animal.

2. The second questionnaire was from the Lexington Attachment to Pets Scale (LAPS) [24]. This is a 23-item scale developed by Johnson et al. in 1992 with the aim of creating a reliable instrument to assess human guardians’ level of emotional attachment to their companion animals. Participants answered the questions by expressing their degree of agreement on a three-point Likert scale (from “strongly agree” to “strongly disagree”) for each of the following factors: general attachment, animals substituting people, and animal rights/welfare. The latter factor assesses the perception of a companion animal’s moral status within the household. In the present study, the focus was only on this scale’s total score (LAPS total alpha = 0.916).

Item example: I love my dog because he/she never judges me.

3. The Pet Bereavement Questionnaire (PBQ) is a questionnaire designed by Hunt and Padilla [7] that consists of 16 items with a four-point Likert response scale (0 = strongly disagree, 3 = strongly agree). The PBQ assesses grief related to the death of a companion animal by considering three different factors: grief (items 2, 3, 5, 7, 10, 12, 15), anger (items 1, 4, 11, 13, 14), and guilt (items 6, 8, 9, 16). Reliability indices were good for this instrument, with Cronbach’s alpha coefficients ranging between 0.67 and 0.86 (PBQ grief alpha = 0.84; PBQ anger alpha = 0.67; PBQ guilt alpha = 0.77; PBQ total alpha = 0.86).

Item example: I am angry with the veterinarian because he/she failed to save my dog.

4. After completing the MDQ, LAPS, and PBQ questionnaires, the study participants filled out the HHHHHMM Quality of Life Scale [21]. This scale is divided into seven criteria which form the acronym of its name: hurt, hunger, hydration, hygiene, happiness, mobility, and more good days than bad. It helps human guardians assess their companion animal’s QoL using a scale from 1 to 10 (1 = unacceptable, 10 = excellent) and make informed decisions about the therapeutic treatments to follow.

Item example: HYGIENE: Did the dog have bedsores?

Finally, the participants were asked to answer four open-ended questions, which were analyzed using a bottom-up thematic analysis [25]. The bottom-up approach involves taking the empirical data as the starting point of the analysis, meaning that no preexisting categories or theoretical concepts are used to analyze the data. Then, the analysts gradually generate categories and themes based on the participants’ words, constantly and recursively checking their adherence to the participants’ perspectives [22,25]. For the present study, two members of the research team independently performed the analysis, which comprised three fundamental phases. During the first phase (creation of codes or coding), analysts created codes using the participants’ words, paying particular attention to ensuring that the codes were strongly linked to the data. During the second phase (creation of categories from codes), the analysts worked directly with the codes and matched them with self-organized categories based on pertinence and similarity criteria [22,25]. During the last phase (thematic generation), the two analysts worked together, and a third member of the research team acted as a judge when any disagreements occurred. The open-ended questions were as follows:

1. Could you describe the changes that occurred in your daily routine, in the short and long term, after the loss of your companion animal?
2. Do you plan to welcome another companion animal into your life, or have you already done so? If yes, can you indicate the reasons for your choice?
3. How would you describe your relationship with the veterinarian who cared for your companion animal, and his/her approach?
4. How did the people closest to you receive the news of your dog’s death? From whom did you receive support? From whom would you have expected more/greater closeness?

### 2.3. Participants

The present study involved individuals residing in Italy who were owners of companion animals that had been affected by chronic, progressive diseases in their later lives. The following inclusion criteria were adopted: having experienced the death of a companion animal and being over 18 years of age. Each eligible participant received a clear explanation of the research objectives and procedure. Participation was voluntary, and participants were given the option of withdrawing at any time. Research participants were recruited through the database of the San Marco Private Veterinary Clinic (Padova District, Veneto Region). A total of 314 adult participants responded positively; among them, 257 were females, 52 were males, and five chose the option “Other” for their gender. Thus, the sample consisted mainly of women (81.8%). The average age of the participants was 44.42 years (SD = 12.15), ranging from 19 to 77 years, and 83.4% were from Northern Italy (262 subjects), while the remaining 16.6% (52 subjects) were from Central and Southern Italy. Out of the total participants, 295 completed the HHHHHMM scale, and 311 completed the PBQ.

The participants were sent a link through which they could access an online survey lasting about 20 min. A total of 242 participants completed the survey. It could be filled out comfortably using either a PC or a smartphone. The research involved a combination of four standardized questionnaires for the factors stated in the objectives.

The study followed the American Psychological Association Ethical Principles as well as the Declaration of Helsinki. Moreover, it was approved by the University of Padova Ethics Committee (Ethical Code AAE32A070588F8595C8F06988799321C).

## 3. Results

### 3.1. Quantitative Analysis

#### 3.1.1. Confirmatory Factor Analysis

The HHHHHMM Quality of Life Scale comprises seven items to measure animals’ wellbeing. A confirmatory factor analysis (CFA) was performed with these seven items to test a one-factor model. The model showed a partially adequate fit, and all parameters were significant (*p* < 0.001) and over 0.40. The standardized parameter estimates for the seven items were 0.48, 0.79, 0.75, 0.43, 0.72, 0.73, and 0.69, respectively. An examination of modification indices indicated two error correlations: one between items 2 and 3 (r = 0.48) and the other between items 3 and 4 (r = 0.49). The two error correlations were included in the modified model because the correlation between these items was supported by the common theme of nutrition for item 2 and item 3 and by the common reference to sores for item 3 and item 4. The new model including these two error correlations showed a good fit. Cronbach’s alpha for the scale was 0.78, indicating good internal consistency. The mean total score was 41.44, and the standard deviation was 15.10. The scale distribution was close to a normal distribution; in fact, it was symmetric but with a slight deviation of the tails (skewness = 0.09 and kurtosis = −0.60). For most human guardians, the dogs’ QoL in the period immediately preceding their death was adequate; for 20% of the participants, it was unacceptable (Table 1).

#### 3.1.2. Correlations between the Study Variables

The scale QoL scale showed (Table 2) only a low negative correlation with anger in the PBQ (r = −0.11, *p* = 0.057), indicating that the human guardians felt more anger when their dogs’ quality of life was poor (Table 2).

The LAPS and PBQ results showed small to moderate positive correlations. In particular, the human guardians experienced greater levels of grief upon their dog’s death when their attachment levels were high (r = 0.47, *p* < 0.001). With regard to human guardians’ characteristics, gender differences were observed only in the LAPS results, and a negative correlation with age was seen in the LAPS and PBQ results. In particular, females showed greater attachment levels than males (t = 3.76, df = 312, *p* < 0.001), and older owners had low attachment levels (r = −0.13, *p* = 0.019) and experienced less distress over their dog’s death (r = −0.16, *p* = 0.005 for grief; r = −0.18, *p* = 0.002 for anger; r = −0.31, *p* < 0.001 for guilt; and r = −0.26, *p* < 0.001 for PBQ total score). Although the results indicated that the human guardian’s gender had no direct effect on the PBQ, the indirect effect of gender on the PBQ, through the LAPS, was always significant (beta = 0.26, *p* = 0.001 for grief; beta = 0.13, *p* = 0.007 for anger; beta = 0.09, *p* = 0.024 for guilt; and beta = 0.21, *p* = 0.002 for PBQ total score). Moreover, the possibility of living in a house with a garden was found to increase dogs’ quality of life (t = 2.33, df = 293, *p* = 0.021) and reduce human guardians’ guilt during bereavement (t = −2.06, df = 309, *p* = 0.040), with respect to those who did not have this opportunity.

Regarding dogs’ characteristics, only one correlation involving sexual status was observed in the LAPS results; human guardians of desexed dogs were found to have greater attachment levels than human guardians of intact dogs (t = 2.11, df = 312, *p* = 0.036). Furthermore, a correlation between dogs’ QoL and the practice of euthanasia was noted; specifically, euthanasia practice was associated with low QoL (t = −2.17, df = 293, *p* = 0.031). Several correlations were observed in the PBQ results: Anger was negatively correlated with dog’s age at death (r = −0.16, *p* = 0.005) and with time spent together by the human guardian and the dog (r = −0.11 *p* = 0.046), indicating that the human guardians experienced more anger when the dog died at a young age and when they had spent less time together. Further, grief was associated with the dog’s age at acquisition, with human guardians experiencing high levels of grief when dogs were young at the time of acquisition (less than three months; t = 2.30, df = 309, *p* = 0.022). Differences in anger and in the PBQ total score were observed based on whether the dog’s death was expected; the human guardians experienced greater distress when their dog’s death was unexpected than when it was expected (t = 3.14, df = 309, *p* = 0.002 for anger; t = 2.00, df = 309, *p* = 0.046 for the PBQ total score). Finally, differences were noted in the guilt factor and PBS total score based on the practice of euthanasia; lower scores were obtained when euthanasia was practiced than when it was not (t = −3.68, df = 309, *p* < 0.001 for guilt; t = −2.25, df = 309, *p* = 0.025 for PBQ total score).

#### 3.1.3. Regression Model to Explain the PBQ

The results of the first regression analysis model confirmed that attachment to one’s dog is always a significant predictor of the PBQ total score and individual factors, with high attachment levels associated with high distress (Table 3).

The human guardian’s age was found to be a significant predictor of the PBQ total score and of guilt; higher ages were associated with lower distress levels. The practice of euthanasia was a significant predictor only of guilt, with low levels of distress experienced when euthanasia was practiced. Dog’s age at acquisition was a significant predictor only of grief, with higher distress levels associated with younger ages. The results of the second regression analysis model showed that three factors had significant interactions with human guardians’ attachment levels: the human guardian’s age, whether the dog’s death was expected, and the dog’s age at death. In particular, the slope analysis of the LAPS for human guardian’s age showed a high impact of LAPS on all PBQ measures for younger human guardians (beta = 0.66, *p* < 0.001 for grief; beta = 0.50, *p* < 0.001 for anger; beta = 0.42, *p* = 0.003 for guilt; beta = 0.66, *p* < 0.001 for total score) than for older guardians (beta = 0.41, *p* = 0.007 for grief; beta = −0.08, *p* = 0.638 for anger; beta = −0.23, *p* = 0.140 for guilt; beta = 0.17, *p* = 0.292 for total score). The LAPS slope analysis also showed a higher impact on grief and PBQ total score when the dog’s death was unexpected (beta = 0.55, *p* < 0.001 for grief; beta = 0.47, *p* < 0.001 for total score) than when it was expected (beta = 0.37, *p* < 0.001 for grief; beta = 0.26, *p* = 0.002 for total score). Finally, the LAPS slope analysis for the dog’s age at death showed a higher impact on anger for younger ages (beta = 0.47, *p* < 0.001) than for older (beta = 0.01, *p* = 0.937).

### 3.2. Qualitative Analysis

The qualitative thematic analysis revealed four major themes: continuing bonds, coping strategies, shared moral values, and perceived support.

#### 3.2.1. Continuing Bonds

This theme indicates that the death of a companion animal affects multiple dimensions of the human guardian. Indeed, the companion animal continues to be seen and heard, perceived through an eternal bond of love, and its death changes the human’s management of time, space, and behavior. This theme is composed of three categories: spirituality, emotions, and behaviors. The first refers to how death shapes the form and intensity of feelings over time. The following is an extract expounding on this category:

“It is difficult to describe what I feel. I lost my little girl, my soulmate. I have two other dogs that I love madly, but none will ever be like my Naira; she was, and she is, full stop. We all suffered at home. Luckily, I had the other dogs and my son near me; otherwise, I would have had a really bad time. For weeks, I kept calling her even after she was gone, looking for her, seeing her shadow in front of the door, preparing her bowls. I have her urn next to me in bed, and she still sleeps there with me and like that forever. My routine has not changed much because, anyway, as I said, I have two other dogs and, to a greater or lesser extent, the things I do with them, I also did with her. The routine I changed most was at the beginning when Raoul, one of the two, went into a crisis. I could no longer keep him alone at home when he went out because he barked with pain. Gradually, our daily life started again, but a huge piece of me left with her. I am sure that my love for her will not end in nothingness; she is definitely with me. Indeed, the night she left, the tap in the bathtub turned on by itself. I will miss her for all my life.”

The “emotions” category refers to how participants have spoken about their intimate feelings after the death of their companion animal. A giant void characterizes all of their words.

“After Mila’s death, I tried to go on taking care of the other dog, Samvise. It was very hard. Sam lost the will to eat. But I accepted her [Mila’s] departure, despite feeling an emptiness inside. Sometimes, I feel like she has been dead for a minute—so much it hurts—sometimes in another life. I just prepare a bowl; I feel like I have nothing to do. I no longer worry about the thunderstorms that used to scare her.”

Finally, the “behaviors” category highlights the short- and long-term behaviors of the human guardians after the death of their companion animal.

“The first few days after her loss, I went into a state of apathy. After dropping the children off at school and kindergarten, I would come home, make myself a cup of tea, and sit on the sofa doing nothing. Slowly, the pain became less intense, and I was able to resume doing small chores. After seven months, it is better, and life is back to its usual pace and rhythm. But I feel her absence, and my children mention her often.”

#### 3.2.2. Coping Strategies

This theme emerged from the participants’ views on the possibility of adopting another companion animal after the loss. Two prevailing strategies were highlighted: (i) the orientation towards a new adoption (this has already happened for some participants) as a way of supporting the management of mourning, or (ii) an open rejection, perceived as permanent or temporary, carried out of respect for the animal that has just died and is considered irreplaceable or out of the need for solitude in the experience of grief. The theme is composed of two categories: desire and rejection. The desire for another companion animal demonstrates a positive orientation characterized by the anticipation of new and positive emotions (happiness and joy) as well as a need to dampen negative emotions (to fill a void and manage psychological distress). The refusal to adopt another companion animal is a negative orientation for various reasons, such as the presence of other animals, the need for family reorganization, a sense of guilt, or the irreplaceability of the dead animal; thus, adopting a new companion animal is not viewed as a way of forming new bonds but as a loss of continuity of the relationship with the dead companion animal. The following extracts from the participants’ answers illustrate desire:

“After a few months, I adopted a new adult dog. My life has always been with animals, and because I could not get over the pain of losing Baloo … a new presence helped me to react … to commit my time to a new creature.”

“Yes, after about a month, another little dog arrived. I was sick; I missed my life partner too much. It did not replace him, but it helped me partly to feel better.”

Rejection is well exemplified by the following extract:

“Not for now. I do not think I would be able to take care of another dog. Plus, I would feel guilty trying to replace him with another animal.”

#### 3.2.3. Shared Moral Values

This theme emerged from the responses describing the emotional consonance that can be generated between a human guardian and a veterinary surgeon when the same values are shared, particularly with respect to the meaning of the bond between an animal and a person and the quality of life and death. This theme is composed of two categories: technical competencies and relational competencies. The category “technical competencies” is exemplified by the following extracts:

“I waited so much during the first visit. After a few minutes, the dog was literally ripped out of my hands. I did not have time to say goodbye to him. Very confusing diagnosis. Initially, it seemed solvable, and then I was told that I would have to put him down. He died on his own the morning of the day I decided to put him down. I hope he was accompanied.”

On the other hand, the category “relational competencies” highlights the importance of creating a professional and personal bond with the human guardian:

“The vet who had been looking after my dog for one year had a lot of patience, especially with me. He was always willing to listen [and was] understanding and attentive to my dog’s needs.”

#### 3.2.4. Perceived Support

This theme refers to a human guardian’s perceived support from others when processing the loss of a companion animal. It emerged from descriptions of the reactions of others to the loss, both positive and negative, as perceived by the respondents, and the respondents’ expectations of support from their close circles (family members, real friends, social friends, and veterinarians). It is composed of two categories: others’ emotional reactions and personal grief. The first category highlights the importance of receiving significant emotional support from one’s social circle. In contrast, the category “personal grief” highlights the fact that the significance of an experience of loss is fundamentally personal. The following two statements exemplify the category “others’ emotional reactions”.

“The people next to me suffered a lot after the loss of the dog, especially my husband. I received support from my sister and niece. I expected more support from friends.”

“The dog died in my arms with all the family people around. It couldn’t have been better than that. All friends who own animals have all been very supportive.”

The following is an example from the category “personal grief”:

“All the people I love have been deeply saddened, but each of us has experienced it alone.”

## 4. Discussion

The present study aimed to validate the HHHHHMM scale, a tool that human guardians can use to assess their companion animals’ QoL, in the Italian context. This instrument can be used to improve bereavement counselling, research, and strategies to help human guardians cope with grief and adjust to the loss of a companion animal. The HHHHHMM (hurt, hunger, hydration, hygiene, happiness, mobility, more good days than bad) Quality of Life Scale is a reliable tool that can be used by guardians of companion animals with a terminal disease. With regard to the research hypotheses, the following arguments can be made:

In relation to the CFA, it has been suggested that RMSEA values less than or equal to 0.05 are good, values between 0.05 and 0.08 are adequate, values between 0.08 and 0.10 are mediocre, and values greater than 0.10 are unacceptable. The comparative fit index (CFI) results in values ranging from zero to one, with higher values indicating a better fit. In addition, SRMR fit values between zero and 0.05 and TLI index values greater than 0.97 indicate a good fit [26]. When using a single-factor model with the total score and the seven items of the HHHHHMM scale, the standardized parameters were all significant (*p* < 0.001), so the model was partially adequate overall.

With regard to the correlations between the instruments and human guardian characteristics, a higher attachment to companion animals was found among women than men in all factors of the LAPS scale. Previous studies have found similar results for displays of affection and caregiving, which have been reported to be more prevalent among women than men [27]. In the present study, it was hypothesized that the LAPS scale measurement items could influence the relationship between human guardians’ gender and the PBQ. Accordingly, independent of the direct effect of gender on the PBQ, an indirect effect—through LAPS—of gender on the PBQ was expected. Thus, although the results indicated that no direct effect between gender and the PBQ exists, the impact that occurs through the LAPS scale is always significant. Women were found to have high levels of attachment to their companion animals; the greater the attachment, the greater the symptoms of grief and suffering experienced following the death of a companion animal. Therefore, women are at greater risk of complicated grief than men after the death of their companion animals. However, due to the insufficient number of male participants, these results cannot be further generalized and need to be studied in more detail.

Negative correlations were found between participant age, attachment, and grief symptoms following the loss of a companion animal. Younger people were found to have higher scores for both attachment and grief symptoms following the death of their dogs, whereas adults and older people had lower scores in both constructs. This finding is consistent with the literature. Indeed, Jarolmen [28] found significant differences in the averages of bereavement symptomatology, with higher values seen among young people than among adults. An individual’s role in the family reaffirms these differences; in fact, children report more feelings of guilt, anger, and distress than parents.

Based on the results, it was hypothesized that the LAPS scale items moderate the effects of human guardians’ age on the PBQ. Furthermore, examining the effect of age at the various levels of the LAPS scale revealed a negative correlation between age and the PBQ at medium and high levels of attachment. This means that younger people exhibit more grief symptoms, such as distress and anger, than older people do. Moreover, at low attachment levels the same negative correlation between age and the PBQ was seen for the factor measuring participants’ feelings of guilt. Young individuals would have few experiences of companion animal loss and may form deep attachment bonds with different companion animals. The lack of experience may make them feel guiltier because of potential idealistic tendencies and the feeling that they should have been able to do more to help their companion animal. In contrast, adult individuals have a lower tendency to feel guilt, perhaps because they spend more time caring for their companion animals than other family members or because their experiences are reflected in more realistic expectations [23].

Family composition was also found to be positively correlated with the LAPS scale’s measurement of attachment to dogs, specifically with the animal rights/welfare factor; it showed that people living alone have a greater tendency to humanize companion animals, specifically dogs, with traits related to social connectedness [29,30]. This may mean that people who do not have a spouse or are not engaged in a romantic relationship feel closer to their companion animals because animals are sources of emotional fulfilment [31]. Another potential reason for this is that keeping a companion animal has a positive effect on happiness and self-esteem and reduces stress, loneliness, and depression [32]. However, it could also impede one’s search for social support from other people, thus having negative effects.

The participants’ anger levels were higher when the companion animal’s death was unforeseen than when they were already aware and thus prepared for the death. These results confirm previous reports in the literature that the unexpected death of a companion animal is associated with high scores for the anger factor of the PBQ [5]. This may be related to the experience of anticipatory grief, which can act as a protective force that allows a person to be better prepared for the death of a loved one, thus reducing the duration and intensity of the postmortem grieving process and preventing complicated grief. Although death is sometimes thought to be more easily accepted when expected because it can facilitate anticipatory grief, when people experience the brevity of the process of death without having the time to prepare in advance, the positive effects of anticipatory grief do not occur. Therefore, low levels of anger are more likely to be related to an individual’s relationship with a veterinarian and the information and preparation obtained. Indeed, when death is anticipated, as in the case of a dog’s terminal illness, the intervention of veterinarians can be very helpful in diminishing human guardians’ feelings of responsibility, validating their decisions, and allowing them to know that they did their best to help their precious companion [33]. The more information a person is given, the better he or she can prepare for what is to come; knowledge of what to expect at the end of a companion animal’s life can decrease fear by containing uncertainty. Therefore, all aspects of impending death should be discussed, including what to expect as the disease progresses and what options are available to manage the disease and provide an adequate QoL for the dog [34].

Participants’ feelings of guilt were lower when death was expected; in these cases, euthanasia was considered a viable option to alleviate the dog’s suffering. In contrast, a good QoL during the dog’s final stages of life made the option of euthanasia more difficult to consider. The dog’s age at death and time spent with the owner were associated with less anger and distress after death, which is consistent with the literature on the MDQ. This could be explained by the fact that a long relationship is able to reduce feelings of anger after death, as has been found in cases of marital bereavement [35]. In light of these results, it was hypothesized that QoL mediates the effects of the dog’s age at death, the presence of a garden at home, and the decision to euthanize based on PBQ scores. The direct effect of the dog’s age at death on the “suffering” factor of the PBQ was found to be significant and negative.

Furthermore, owning a house with a garden was found to have a significant positive impact on the dog’s quality of life, while the euthanasia was significantly negatively correlated with QoL. The direct effect of QoL on the PBQ factor “anger” was significant and negative. This means that a dog’s quality of life was considered good if the human guardian had a house with a garden; in turn, providing their dogs with a satisfactory QoL led to the human guardians reporting fewer feelings of anger at the time of death. Human guardians who assessed their dog’s quality of life as unacceptable and chose to alleviate its suffering by administering euthanasia reported fewer feelings of anger after the animal’s death. This may be explained by the fact that many owners may not have the time to prepare for a companion animal’s death or may not have received adequate support [23]. Notably, although it does not prevent the effects of depression, receiving detailed information about the companion animal’s health condition from a veterinarian can reduce both the anger and guilt felt by human guardians [5].

## 5. Conclusions

The aim of the present study was to validate, in the Italian context, an easy-to-use scale for assessing the quality of life of companion animals with terminal illnesses. Companion animal loss can elicit significant grief responses comparable to those caused by the death of a loved one. The main symptoms related to grief for humans, namely guilt, grief, anger, and intrusive thoughts, often occur after the loss of a companion animal. Therefore, people may be at risk of complicated grief responses to the death of their companion animals. In addition, grief resulting from the death of a companion animal is among the forms of grief that are delegitimized, causing a lack of social support for the bereaved. Veterinarians need to be aware of the peculiarities of this type of grief to readily accommodate, support, and acknowledge the legitimacy of human guardians’ suffering. Finally, although this was limited to the Italian context, similar results would likely emerge from the use of the scale in social and cultural contexts wherein the domestic relationship between companion animals and human guardians is diffused.

The HHHHHMM Quality of Life scale is a reliable tool that can be used by individuals to make end-of-life decisions for their companion animal, preventing regret and guilt after the companion animal’s death. This tool can be used to implement coping strategies based on rationalization to improve the well-being and resilience of individuals and families. It is of paramount importance to provide psychological counselling during decision-making processes related to chronic illnesses and end-of-life care for companion animals. There is growing awareness in Italy of the need to pay more attention to companion animal bereavement and end-of-life issues in veterinary medicine, given the important role that companion animals play in people’s lives.

However, this research has some limitations. First, the sample of respondents was decidedly biased in favor of the female gender. The generalizability of the results is limited because the sample in this study consisted mainly of middle-aged, married, and well-educated women. In the future, it will be necessary to recruit a balanced number of male and female participants. As with all surveys that only include people who use the internet, this study may have had a sampling bias compared to the general population, as internet use is not uniform across different demographic, cultural, and geographical groups. Finally, the instruments were used retrospectively to measure participants’ attachment and grief symptoms in relation to past events.

## Figures and Tables

**Table 1:** Results of the confirmatory factor analysis of QoL (N = 295).

Model	Chi-Square	df	*p*-Value	Chi-Square/df	RMSEA	SRMR	CFI	TLI
one-factor original	88.05	14	<0.001	6.29	0.13	0.08	0.97	0.96
one-factor modified	11.00	12	0.53	0.92	0.00	0.03	1.00	1.00

**Table 2:** Correlations between the study variables (N = 295).

Variables	1	2	3	4	5	6
1. QoL Total score	-					
2. LAPS Total score	0.00	-				
3. PBQ Grief	−0.04	0.47 ***	-			
4. PBQ Anger	−0.11 ~	0.23 ***	0.52 ***	-		
5. PBQ Guilt	−0.04	0.17 **	0.38 ***	0.52 ***	-	
6. PBQ Total score	−0.07	0.39 ***	0.84 ***	0.81 ***	0.76 ***	-

~ *p* < 0.10; ** *p* < 0.01; *** *p* < 0.001.

**Table 3:** Regression analysis results for the PBQ.

	PBQ Grief		PBQ Anger		PBQ Guilt		PBQ Total Score	
	Model1	Model2	Model1	Model2	Model1	Model2	Model1	Model2
Main effect of variables								
Owner’s age	−0.08	−0.06	−0.11~	−0.12 *	−0.27 ***	−0.27 ***	−0.18 **	−0.17 **
House with garden (1 = Yes, 0 = No)	−0.04	−0.04	−0.04	−0.03	−0.10 ~	−0.10 ~	−0.07	−0.07
Early dog’s age at acquisition (1 = Yes, 0 = No)	0.14 **	0.15 **	0.08	0.09	−0.02	−0.02	0.09 ~	0.10 ~
Dog’s death was expected (1 = Yes, 0 = No)	0.00	0.02	−0.12 ~	−0.08	−0.06	−0.03	−0.06	−0.03
Dog was euthanized (1 = Yes, 0 = No)	0.00	0.01	−0.01	0.01	−0.15 **	−0.14 *	−0.06	−0.05
Dog’s age at death	−0.06	−0.06	−0.11 ~	−0.09	0.10~	0.11 ~	−0.03	−0.02
QoL Total score	−0.03	−0.03	−0.11 ~	−0.09	−0.02	−0.01	−0.06	−0.05
LAPS Total score	0.45 ***	0.54 ***	0.20 ***	0.21	0.13 *	0.33 *	0.35 ***	0.48 **
Interactions with LAPS								
Owner’s age by LAPS		−0.13 *		−0.18 **		−0.14 *		−0.18 **
House with garden by LAPS		0.11		0.01		−0.08		0.03
Early dog’s age at acquisition by LAPS		0.02		0.04		−0.03		0.01
Dog’s death was expected by LAPS		−0.17 *		−0.06		−0.09		−0.14 ~
Dog was euthanized by LAPS		−0.10		−0.01		−0.07		−0.08
Dog’s age at death by LAPS		0.09		−0.12 ~		−0.04		−0.01
QoL Total score by LAPS		0.03		−0.04		−0.06		−0.02
R-square	26%	29%	13%	18%	16%	19%	22%	27%

The values reported in the table are standardized regression coefficients (beta). ~ *p* < 0.10; * *p* < 0.05; ** *p* < 0.01; *** *p* < 0.001.

## Data Availability

The data presented in this study are available on request from the corresponding author. The data are not publicly available due to privacy.

## References

[1] Alderton D. (2011). Animal Grief-How Animals Mourn.

[2] Testoni I., Antonellini M., Ronconi L., Biancalani G., Neimeyer R.A. (2022). Spirituality and Meaning-Making in Bereavement: The Role of Social Validation. J. Loss Trauma.

[3] Pirrone F., Pierantoni L., Mazzola S.M., Vigo D., Albertini M. (2015). Owner and animal factors predict the incidence of, and owner reaction toward, problematic behaviors in companion dogs. J. Vet. Behav..

[4] Testoni I., De Cataldo L., Ronconi L., Colombo E.S., Stefanini C., Dal Zotto B., Zamperini A. (2019). Pet Grief: Tools to Assess Owners’ Bereavement and Veterinary Communication Skills. Animals.

[5] Testoni I., De Cataldo L., Ronconi L., Zamperini A. (2017). Pet Loss and Representations of Death, Attachment, Depression, and Euthanasia. Anthrozoös.

[6] Gerwolls M.K., Labott S.M. (1994). Adjustment to the Death of a Companion Animal. Anthrozoös.

[7] Hunt M., Padilla Y. (2006). Development of the pet bereavement questionnaire. Anthrozoös.

[8] Thomas J.C., Sours T. (2007). Multiple lacerations of the heart: When grief accumulates. Christ. Couns. Connect. Summer.

[9] Cowles K.V. (2016). The death of a pet: Human responses to the breaking of the bond. Pets and the Family.

[10] Wrobel T.A., Dye A.L. (2003). Grieving Pet Death: Normative, Gender, and Attachment Issues. OMEGA-J. Death Dying.

[11] Packman W., Carmack B.J., Ronen R. (2012). Therapeutic Implications of Continuing Bonds Expressions following the Death of a Pet. OMEGA-J. Death Dying.

[12] Cordaro M. (2012). Pet Loss and Disenfranchised Grief: Implications for Mental Health Counseling Practice. J. Ment. Health Couns..

[13] Blazina C. (2011). Life after loss: Psychodynamic perspectives on a continuing bonds approach with “pet companion”. The Psychology of the Human-Animal Bond.

[14] Knesl O., Hart B.L., Fine A.H., Cooper L., Patterson-Kane E., Houlihan K.E., Anthony R. (2017). Veterinarians and Humane Endings: When Is It the Right Time to Euthanize a Companion Animal?. Front. Vet. Sci..

[15] Leary S., Underwood W., Anthony R., Cartner S., Grandin T., Greenacre C., Gwaltney- Brant S., McCrackin M.A., Meyer R., Miller D. (2020). AVMA Guidelines for the Euthanasia of Animals, 2020 Edition.

[16] Zarit S.H., Zarit J.M. (1986). Dementia and the family: A stress management approach. Clin. Psychol..

[17] Spitznagel M.B., Jacobson D.M., Cox M.D., Carlson M.D. (2017). Caregiver burden in owners of a sick companion animal: A cross-sectional observational study. Vet. Rec..

[18] Christiansen S.B., Kristensen A.T., Sandøe P., Lassen J. (2013). Looking after Chronically III Dogs: Impacts on the Caregiver’s Life. Anthrozoös.

[19] Villalobos A.E. (2011). Assessment and Treatment of Nonpain Conditions in Life-Limiting Disease. Vet. Clin. N. Am. Small Anim. Pract..

[20] Villalobos A.E. (2019). Supportive Care for People with Disabilities as Working Partnerships with Their Assistance Dogs Are Ending: A Perspective from Veterinary Oncology. Front. Vet. Sci..

[21] Villalobos A.E. (2011). Quality-of-Life Assessment Techniques for Veterinarians. Vet. Clin. N. Am. Small Anim. Pract..

[22] Testoni I., De Vincenzo C., Zamperini A., Kõlves A., Sisask M., Värnik P., Värnik A., De Leo D. (2021). The words to say it—Qualitative suicide research. Advancing Suicide Research.

[23] Uccheddu S., De Cataldo L., Albertini M., Coren S., Da Graça Pereira G., Haverbeke A., Mills D.S., Pierantoni L., Riemer S., Ronconi L. (2019). Pet Humanisation and Related Grief: Development and Validation of a Structured Questionnaire Instrument to Evaluate Grief in People Who Have Lost a Companion Dog. Animals.

[24] Johnson T.P., Garrity T.F., Stallones L. (1992). Psychometric Evaluation of the Lexington Attachment to Pets Scale (Laps). Anthrozoos.

[25] De Vincenzo C., Serio F., Franceschi A., Barbagallo S., Zamperini A. (2022). A “Viral Epistolary” and Psychosocial Spirituality: Restoring Transcendental Meaning during COVID-19 through a Digital Community Letter-Writing Project. Pastor. Psychol..

[26] Schermelleh-Engel K., Moosbrugger H., Müller H. (2003). Evaluating the Fit of Structural Equation Models: Tests of Significance and Descriptive Goodness-of-Fit Measures. Methods Psychol. Res. Online.

[27] Prato-Previde E., Fallani G., Valsecchi P. (2006). Gender Differences in Owners Interacting with Pet Dogs: An Observational Study. Ethology.

[28] Jarolmen J. (1998). A comparison of the grief reaction of children and adults: Focusing on pet loss and bereavement. OMEGA-J. Death Dying.

[29] Epley N., Waytz A., Akalis S., Cacioppo J.T. (2008). When We Need A Human: Motivational Determinants of Anthropomorphism. Soc. Cogn..

[30] Haslam N. (2022). Dehumanization and the lack of social connection. Curr. Opin. Psychol..

[31] Albert A., Bulcroft K. (1987). Pets and Urban Life. Anthrozoös.

[32] Le Roux M.C., Wright S. (2020). The Relationship Between Pet Attachment, Life Satisfaction, and Perceived Stress: Results from a South African Online Survey. Anthrozoös.

[33] Kerrigan L. (2014). Anticipating grief—The role of pre-euthanasia discussions. Vet. Nurse.

[34] Cox S., Gardner M., McVety D. (2017). Anticipatory Grief and Preparation for Pet Loss. Treatment and Care of the Geriatric Veterinary Patient.

[35] Kim S.H. (2009). The influence of finding meaning and worldview of accepting death on anger among bereaved older spouses. Aging Ment. Health.

